# Adult aplastic anemia in Thailand: incidence and treatment outcome from a prospective nationwide population-based study

**DOI:** 10.1007/s00277-021-04566-0

**Published:** 2021-07-16

**Authors:** Lalita Norasetthada, Somchai Wongkhantee, Jindaratn Chaipokam, Kanyaporn Charoenprasert, Suporn Chuncharunee, Ponlapat Rojnuckarin, Chittima Sirijerachai, Wanchai Wanachiwanawin, Surapol Issaragrisil, for the Thai Aplastic Anemia Study Group

**Affiliations:** 1 Division of Hematology, Department of Internal Medicine, Faculty of Medicine, Chiang Mai University, Chiang Mai, Thailand; 2 Department of Internal Medicine, Khon Kaen Regional Hospital, Khon Kaen, Thailand; 3 Department of Internal Medicine, Sakon Nakhon Hospital, Sakon Nakhon, Thailand; 4 Department of Internal Medicine, Sisaket Hospital, Sisaket, Thailand; 5 Division of Hematology, Department of Medicine, Faculty of Medicine Ramathibodi Hospital, Mahidol University, Bangkok, Thailand; 6 Division of Hematology, Department of Medicine, Faculty of Medicine, Chulalongkorn University, Bangkok, Thailand; 7 Division of Hematology, Department of Internal Medicine, Faculty of Medicine, Khon Kaen University, Khon Kaen, Thailand; 8 Division of Hematology, Department of Medicine, Faculty of Medicine Siriraj Hospital, Mahidol University, Bangkoknoi, Bangkok, 10700 Thailand

**Keywords:** Aplastic anemia, Population-based study, Incidence, Rabbit anti-thymocyte globulin, Anabolic steroids

## Abstract

The incidence and outcomes of aplastic anemia (AA) in Asia remain limited. This study aimed to explore the incidence and outcomes of patients with adult AA across the country of Thailand. This is a prospective multi-center nationwide population-based observational study of AA patients aged at least 15 years old, diagnosed from August 2014 to July 2016, with a longitudinal follow-up period over 2 years. There were 348 newly diagnosed adult AA patients during the enrollment period, giving an annual incidence of 4.6 per million. The incidence of severe (SAA) and very severe aplastic anemia (VSAA) (3.8 per million) was higher than non-severe AA (NSAA, 0.8 per million). The peak incidence was observed in the patients aged from 80 to 89 years old (14.4 per million). The 2-year overall survival (OS) in NSAA, SAA, and VSAA were 65.5%, 49.3%, and 20.1%, respectively (*P* < 0.001). With regard to the response to immunosuppressive therapy, the overall response rate (ORR) in SAA/VSAA treated with rabbit anti-thymocyte globulin with/without cyclosporin A (rATG ± CsA) were significantly superior to those treated with CsA alone, or anabolic steroids (44.4% vs 36.4% and 31.2%, respectively, *P* < 0.001). The 2-year OS in SAA/VSAA treated with rATG ± CsA, CsA, and anabolic steroids were 54.8%, 54.5%, and 37.6% (*P* = 0.037), respectively. The incidence of adult AA in Thailand is higher than those in Western countries, and the peak incidence is in the elderly. rATG ± CsA provided a better response than anabolic steroids, translating to the superior survival in SAA/VSAA treated with rATG ± CsA.

**Supplementary Information:**

The online version contains supplementary material available at 10.1007/s00277-021-04566-0.

## Introduction

The incidence of aplastic anemia (AA) varied worldwide. The estimated annual incidence in Western countries was 1.5–2.3 per million [1–5], while it was 2–3 times higher in Asia (3.0–7.5 per million) [6–9]. The explanation for these differences is unknown; however, it may be related to genetic or environmental factors. The relationship between AA and environmental exposure including certain medications, benzene, contaminated water sources, animal fertilizers, and pesticides was previously reported [10, 11].

Immunosuppressive therapy (IST) is recommended for the treatment of SAA patients who are not eligible for hematopoietic stem cell transplantation (HSCT) [12, 13]*.* Although horse ATG (hATG) gives better responses over rabbit ATG (rATG) as first-line therapy [13], rATG is widely used as initial therapy in Europe and Asia due to unavailability of the hATG. Recent multi-national studies of rATG in Europe and Asia showed 1-year response rates of 63–65% with favorable survival outcomes (2-year overall survival 79.0–86.3%) [14–16]. Anabolic steroids were used as an alternative therapy for those unaffordable or unsuitable for HSCT or IST. To understand the current situation of adult AA in Thailand, the annual incidence of AA across the country and the treatment responses as well as survival outcome according to patient’s characteristics and treatment modalities were studied.

## Methods

### Study design and patients

This study was a multi-center prospective observational study. Newly diagnosed adult AA patients from 30 referral centers nationwide (aged at least 15 years) were enrolled during the period from August 1, 2014, to July 31, 2016. Criteria for diagnosis of AA included bone marrow hypocellularity with at least two of the following: anemia with corrected reticulocyte count ≤ 1%, absolute neutrophil count (ANC) ≤ 1.5 × 10^9^/L, or platelet count ≤ 50 × 10^9^/L. Patients with myelodysplastic syndrome (MDS) or classic paroxysmal nocturnal hemoglobinuria and chemotherapy-induced bone marrow suppression were excluded. Patients with chromosomal abnormalities were also excluded. Those with nationalities other than Thai were excluded. All enrolled patients were reassessed for definite diagnosis by the central review committee. Treatment of choice was given at the discretion of the treating physicians, depending on the patient’s eligibility and accessibility to treatment. The research proposal and all subsequent amendments were approved by the local Ethics Committee at each study site. The study was conducted in accordance with ethical standards of the responsible committee on human experimentation and with the Helsinki Declaration of 1975, as revised in 2008. All patients provided written informed consent.

### Definition

SAA has at least 2 of the following: reticulocyte count ≤ 1%, ANC ≤ 0.5 × 10^9^/L, platelet count ≤ 20 × 10^9^/L, and hypocellular bone marrow with ≤ 25% cellularity. VSAA fulfills the definition of SAA but with ANC ≤ 0.2 × 10^9^/L. Those who do not meet the criteria for SAA/VSAA are NSAA.

### Responses

Responses to treatment were evaluated at each visit every 3 months. In those who switched to subsequent therapy, the responses were reassessed to evaluate the efficacy of the second-line treatment. Criteria for responses were based on the British Committee for Standard Haematology (BCSH) guideline [12, 17]. Briefly, the response criteria were as follows: complete response (CR): normal hemoglobin (Hb) for age and gender with ANC ≥ 1.5 × 10^9^/L and platelet ≥ 150 × 10^9^/L; partial response (PR): transfusion independence and no longer met the criteria for severe disease, and no response (NR): not achieving CR/PR.

The response criteria for NSAA were similar to SAA for CR and NR, whereas the criteria for PR were transfusion independence (if previously dependent) or doubling or normalization of at least one lineage or increase of baseline Hb ≥ 3 g/dL, ANC ≥ 0.5 × 10^9^/L (if initially < 0.5 × 10^9^/L), and platelet ≥ 20 × 10^9^/L (if initially < 20 × 10^9^/L).

### Statistical analyses

**Fig. 1:**
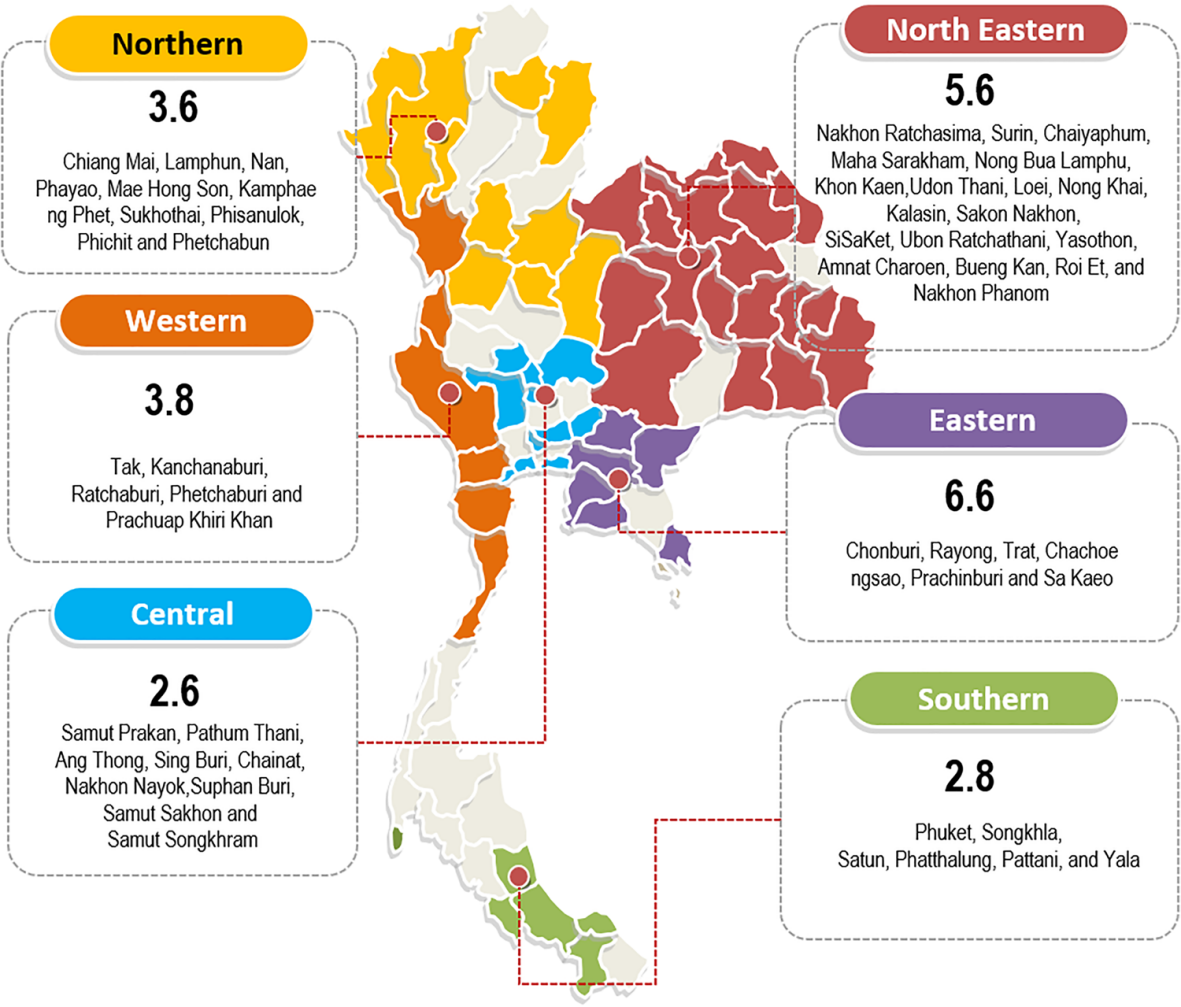
Incidence by geographical regions. Shaded areas represent provinces of the catchment areas where the provincial territories were under the service coverage by the study sites. Numbers indicate age-standardized incidence (per million populations per year) of each geographical region

The incidence rate was calculated by the number of newly diagnosed AA patients divided by the number of inhabitants within the respective catchment areas. All eligible cases were recruited from almost all hospitals over the country. We excluded hospitals in which there were no hematologists. The catchment areas were defined to only the provinces with the hematology services (Fig. 1). Only patients who had lived at least 6 months in the catchment area were included into the incidence calculation. Population numbers were collected from the national database provided by the Strategy and Planning Division, Office of Permanent Secretary, Ministry of Public Health (http://bps.moph.go.th/new_bps/). Direct age standardization was presented as previously described [18].

Comparisons of data were performed using t-test or Wilcoxon rank-sum test or χ^2^, depending on variable types and data distribution. OS was analyzed using Kaplan–Meier plots. Cox regressions were used to estimate hazard ratios among given subgroup stratifications. Treatment responses to immunosuppressive therapy were pre-defined as previously described criteria [17]. The statistical analyses were performed using SAS 9.4.

## Results

A total of 348 patients were enrolled. Twenty-six patients were excluded for the incidence calculation because they had lived in the catchment area less than 6 months. Six patients were excluded from the treatment response analyses due to inadequate collected data.

### Incidence of AA

There were 322 patients with newly diagnosed adult AA during the 2-year periods, which yielded an overall annual incidence of 4.6 per million. The annual incidence of SAA/VSAA was 3.8 per million, higher than that of NSAA (0.8 per million with a ratio of 4.6:1). Forty-five patients had VSAA, giving the annual incidence of 0.6 per million. The incidence was high in older patients with the peak of 14.4 per million in patients aged from 80 to 89 years old (Table 1). It was lower in patients aged less than 50 (1.2–2.3 per million). There was a slightly higher incidence in men than women (4.8 vs 4.0 per million). Age-standardized incidence rates (ASRs) were reported by geographical regions, which showed the highest incidences in eastern and north-eastern areas with ASRs of 6.6 and 5.6 per million, respectively. Variation in incidence rate was observed across the country, ranging from 2.6 to 6.6 per million (Fig. 1, Table S1).

**Table 1:** Incidence of AA in Thailand (2014–2016)

Population		Rate per million inhabitants per year					
		Non-severe AA		Severe and very severe AA		Overall	
		(*N* = 57)		(*N* = 265)		(*N* = 322†)	
		N	Incidence	N	Incidence	N	Incidence
Overall	69,846,949	57	0.8	265	3.8	322	4.6
Age group							
- 15–19	6,157,151	3	0.5	11	1.8	14	2.3
- 20–29	12,510,564	5	0.4	21	1.7	26	2.1
- 30–39	13,663,819	2	0.2	14	1.0	16	1.2
- 40–49	14,097,108	5	0.3	28	2.0	33	2.3
- 50–59	11,244,003	1	1.1	58	5.2	70	6.2
- 60–69	6,910,825	1	2.5	74	10.7	91	13.2
- 70–79	3,545,979	7	2.0	42	11.8	49	13.8
- 80–89	1,426,750	5	3.4	16	10.9	21	14.4
- 90–99	227,726	1	4.4	1	4.4	2	8.8
Gender							
- Male	34,134,691	23	0.7	147	4.3	170	5.0
- Female	35,712,258	34	0.9	118	3.3	152	4.3

^†^Excluding 26 patients due to their residential locations situated outside the pre-defined geographical catchment areas

### Patients’ characteristics

With regard to the severity, there were 45 patients (13.1%), 238 (69.5%), and 59 (17.2%) with VSAA, SAA, and NSAA, respectively (Table S2). The majority of the patients (82.6%) were considered at least severe. The median age was 59 years old (range, 15–93). Seventy-two percent (72%) of them aged at least 50 years. Patients with VSAA had more severe manifestation: i.e., infections (44.4% in VSAA vs 16.8% in SAA vs 11.9% in NSAA, *P* = 0.005) and bleeding (80.0% in VSAA vs 59.2% in SAA vs 40.7% in NSAA, *P* = 0.0003). Frequent environmental exposures were agriculture pesticides (*n* = 60), certain medications previously reported as possible cause of AA within 6 months (*n* = 32), ducks/geese, and animal fertilizer (*n* = 15).

### Treatment modalities

Of 283 SAA/VSAA patients, only 3 patients underwent HSCT, 143 received rATG ± CsA, 12 CsA only, 102 anabolic steroids as the frontline therapy, and supportive treatment in 23 patients (Table 2). For rATG treatment, it was frontline treatment in 143 patients and additionally as second line in 10 patients. Twenty patients underwent second rATG treatment after failure or relapse to the first rATG.

**Table 2:** Treatment modalities according to the frontline and subsequent treatments for patients with SAA and VSAA

*n* = 280^‡^	Frontline treatment, n (%)		Subsequent treatment^†^, n (%)	
	rATG ± CsA*	143 (51.1)	rATG ± CsA	20 (14.0)
			Anabolic steroids	20 (14.0)
			CsA-based	5 (3.5)
	Anabolic steroids	102 (36.4)	rATG ± CsA	9 (8.8)
			CsA-based	1 (1.0)
	CsA-based^#^	12 (4.3)	rATG ± CsA	1 (8.3)
			Anabolic steroids	1 (8.3)
	No specific treatment	23 (8.2)	-	

^‡^Did not include 3 patients who underwent HSCT

^*^rATG ± CsA: rATG with or without CsA; rATG with CsA (n = 123); rATG without CsA (n = 20)

^#^CsA-based: CsA contained treatment without ATG

^†^Subjects without subsequent treatment were not included

*rATG*, rabbit anti-thymocyte globulin; *CsA*, cyclosporin A

One hundred thirty-seven (137) patients with SAA/VSAA did not receive frontline rATG. The reasons included patient conditions not suitable for IST (such as active infections and comorbidities, *n* = 58), inaccessibility to rATG (*n* = 35), concerns regarding medical facility (*n* = 17), and other reasons (*n* = 27).

In 59 NSAA patients, the majority of them (*n* = 49, 83.1%) received anabolic steroids as frontline therapy, while 8 received supportive treatment. The other 2 patients received CsA.

### Treatment response in patients with SAA/VSAA

Two-hundred eighty patients were evaluated for their treatment responses. Patients with VSAA had inferior ORR than those with SAA (15.6% vs 40.0%, *P* = 0.002) (Table S4A). The ORR among patients treated with rATG ± CsA (44.4%) was significantly superior than those treated with CsA-based treatment (36.4%) and anabolic steroids (31.2%) (P < 0.001). The ORR of first treatment with rATG ± CsA (*n* = 153) at 3, 6, 12, and 24 months were 17.6% (95% CI, 12.0–24.6), 30.1% (95% CI, 22.9–38.0), 34.0% (95% CI, 26.5–42.1), and 37.9% (95% CI, 32.0–46.1), respectively (Table 3). The ORR among evaluable patients were 23.9%, 43.8%, 68.4%, and 89.2% at 3, 6, 12, and 24 months, respectively. Among 20 patients who received second rATG ± CsA, 1-year ORR was 25% (95% CI, 6.0–44.0). From multivariate analysis, anabolic steroids had lower ORR than rATG ± CsA with an odds ratio (OR) of 0.57 (95% CI, 0.33–0.98, *P* = 0.04) (Table S4A).

**Table 3:** Response rates over time after rATG ± CsA and anabolic steroids treatments in SAA/VSAA

	Month 3	Month 6	Month 12	Month 24
First ATG ± CsA† (*N* = 153)				
ORR, % (95% CI)	17.6 (12.0–24.6)	30.1 (22.9–38.0)	34.0 (26.5–42.1)	37.9 (30.2–46.1)
CR, n (%)	0 (0)	3 (2.0)	3 (2.0)	8 (5.2)
PR, n (%)	27 (17.6)	43 (28.1)	49 (32.0)	50 (32.7)
Not evaluable* n (%)	40 (26.1)	48 (31.4)	77 (50.3)	88 (57.5)
ORR in evaluable patients** no. (%)	27/113 (23.9)	46/105 (43.8)	52/76 (68.4)	58/65 (89.2)
Second ATG ± CsA (*N* = 20)				
ORR, % (95% CI)	10.0 (0–8–14)	10.0 (0–8–14)	25 (6.0–44.0)	35 (14.1–55.9)
CR, n (%)	0 (0.0)	0 (0.0)	1 (5.0)	1 (5.0)
PR, n (%)	2 (10.0)	2 (10.0)	4 (20.0)	6 (30.0)
Not evaluable* n (%)	0 (0.0)	3 (15.0)	5 (25.0)	8 (40.0)
Anabolic steroids (*N* = 93)				
ORR, % (95% CI)	11.8 (6.1–20.2)	18.3 (11.0–27.6)	28.0 (19.1–38.2)	21.5 (13.7–31.2)
CR, n (%)	0 (0.0)	0 (0.0)	5 (5.4)	3 (3.2)
PR, n (%)	11 (11.8)	17 (18.3)	21 (22.6)	17 (18.3)
Not evaluable* n (%)	37 (39.8)	47 (50.5)	52 (55.9)	71 (76.3)

^†^Including patients receiving first ATG ± CsA as initial (*n* = 143) and subsequent treatment (*n* = 10)

^*^Not evaluable due to lost to follow-up or death

^**^Excluding non-evaluable patients at each time point

*rATG*, rabbit anti-thymocyte globulin; *CsA*, cyclosporin A; *ORR*, overall response rate; *CR*, complete response; *PR*, partial response

**Fig. 2:**
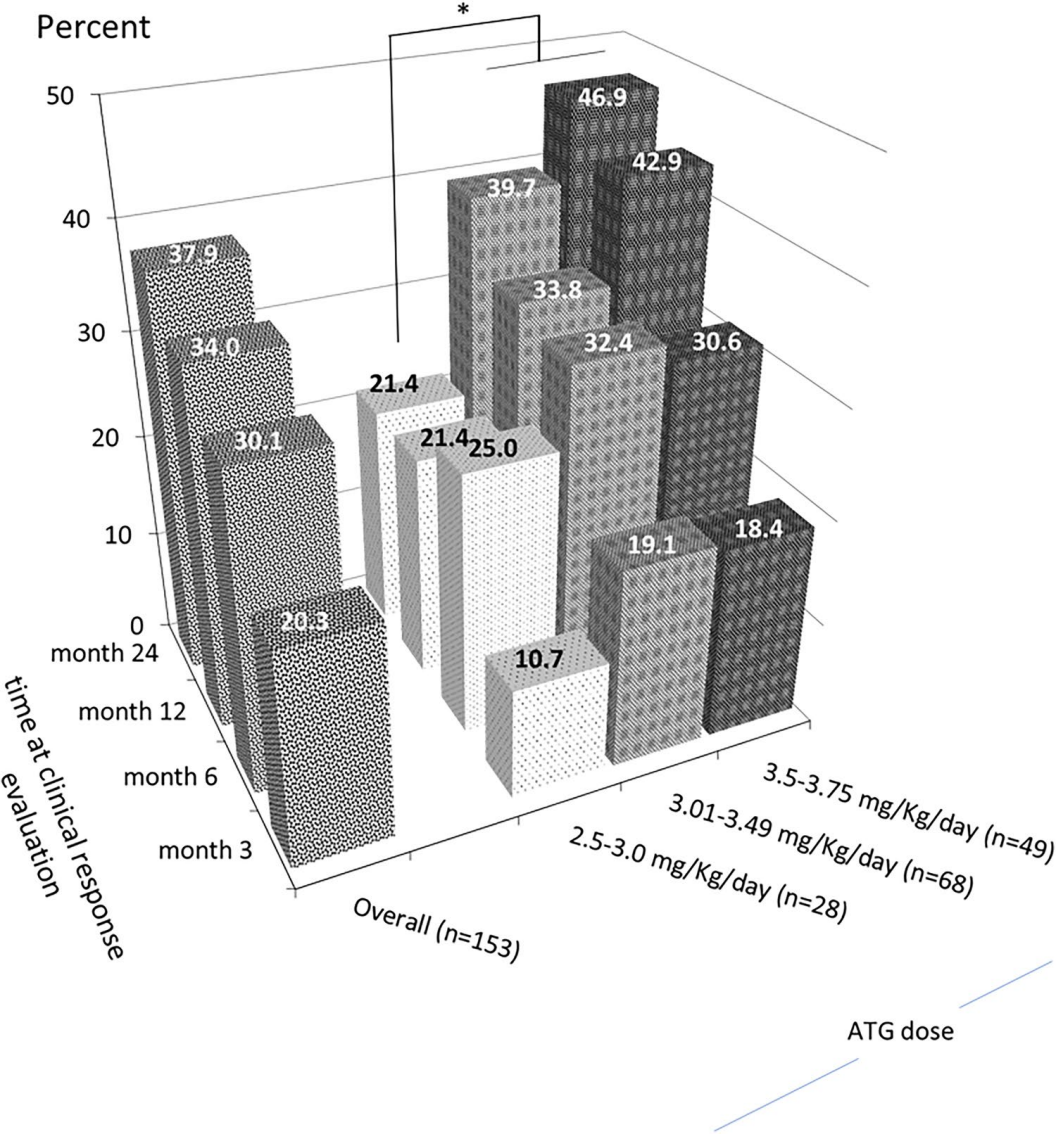
Overall response rate to rATG ± CsA in SAA/VSAA at each time point according to rATG dosage. Y-axis represented the overall response rate. Z-axis represented time point of response evaluation after treatment with rATG. X-axis represented rATG dosage range (mg/kg/day). * denotes statistical significance with *P*-value of 0.038

The ORR to rATG ± CsA was lower in patients older than 60 years (32.0% vs 50.5% for ≤ 60 years, *P* = 0.02) while patients with VSAA had a trend of a poorer response than those with SAA (23.5% vs 47.1%, *P* = 0.06) (Table S5). At 24 months, the rATG dose range of 3.0–3.5 mg/kg/day (39.7%) and 3.5–3.75 mg/kg/day (46.9%) provided a better response rate than that of the lower dose range of 2.5–3.0 mg/kg/day (21.4%, *P* = 0.03) (Fig. 2). Nevertheless, the serious uncommon adverse events of the higher dose range of 3.5–3.75 mg/kg/day were observed including hepatotoxicity (*n* = 2), acute renal failure (*n* = 2), and cardiac arrest (*n* = 1) (Table S6).

### Treatment response in patients with NSAA

Of 49 patients with NSAA who were treated with anabolic steroids, 20 patients (40.8%) responded to treatment with a CR rate of 6.1% (*n* = 3). The ORR of anabolic steroids at 3, 6, 12, and 24 months were 28.6% (95% CI, 16.6–43.3%), 34.7% (95% CI, 21.7–49.6%), 40.8% (95% CI, 27.0–55.8%), and 34.7% (95% CI, 21.7–49.6%), respectively (Table S3).

### Survival outcome of all patients

**Fig. 3:**
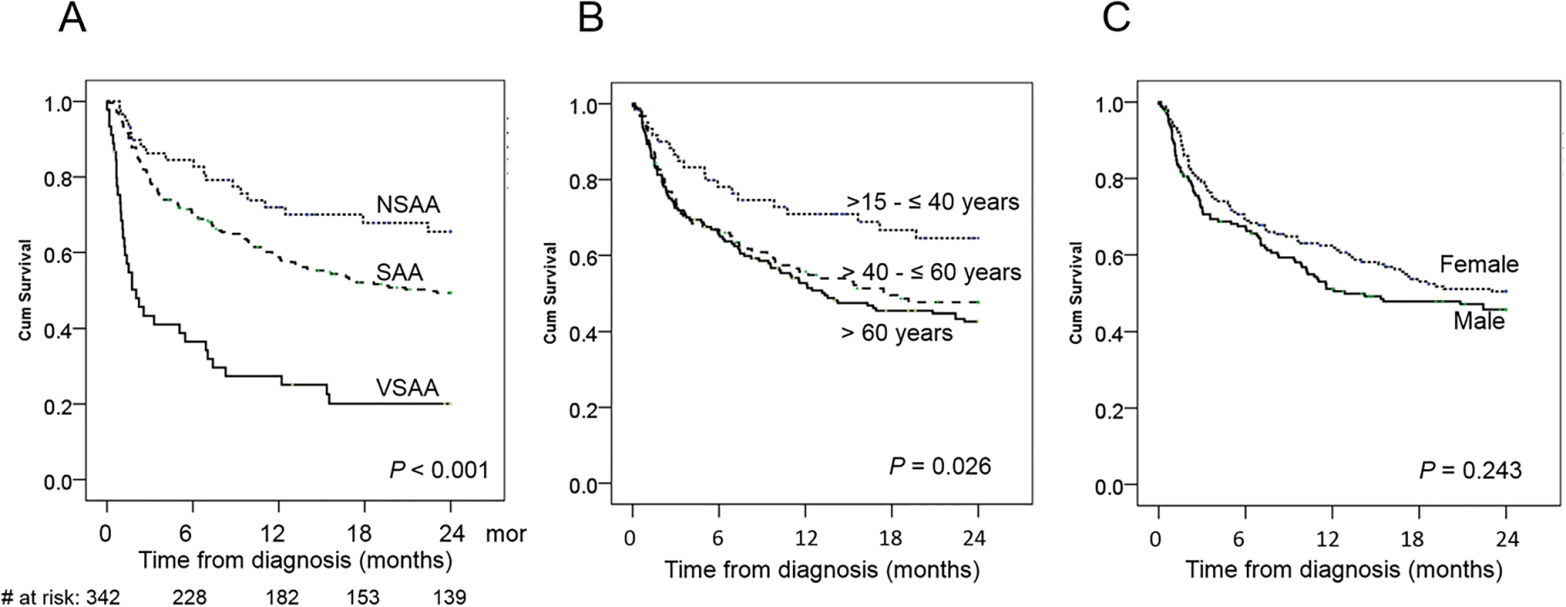
Kaplan–Meier plots for overall survivals of aplastic anemia patients (*n* = 342) according to the severity of disease (**A**), patient’s age groups (**B**), and gender (**C**)

Among 342 patients with AA, the median OS was 19.2 months (95% CI, 12.9 months-not reached) with a 2-year OS of 48.1% (95% CI, 45.3–50.4). When stratified by the disease severity, the 2-year OS for NSAA, SAA, and VSAA were 65.5% (95% CI, 59.0–72.0), 49.3% (95% CI, 46.0–52.6), and 20.1% (95% CI, 14.0–26.2), respectively (*P* < 0.001) (Fig. 3A). The 2-year OS for patients aged 15–40, 41–60, and > 60 years were 64.5% (95% CI, 58.0–71.0), 47.7% (95% CI, 43.1–49.3), and 42.6% (95% CI, 38.6–46.6) (*P* = 0.02), respectively (Fig. 3B). There was no significant difference in survivals between gender with 2-year OS in males and female of 50.4% (95% CI, 46.5–54.3) and 45.7% (95% CI, 41.7–49.7), respectively (*P* = 0.24) (Fig. 3C).

### Survival outcome of patients with SAA/VSAA

**Fig. 4:**
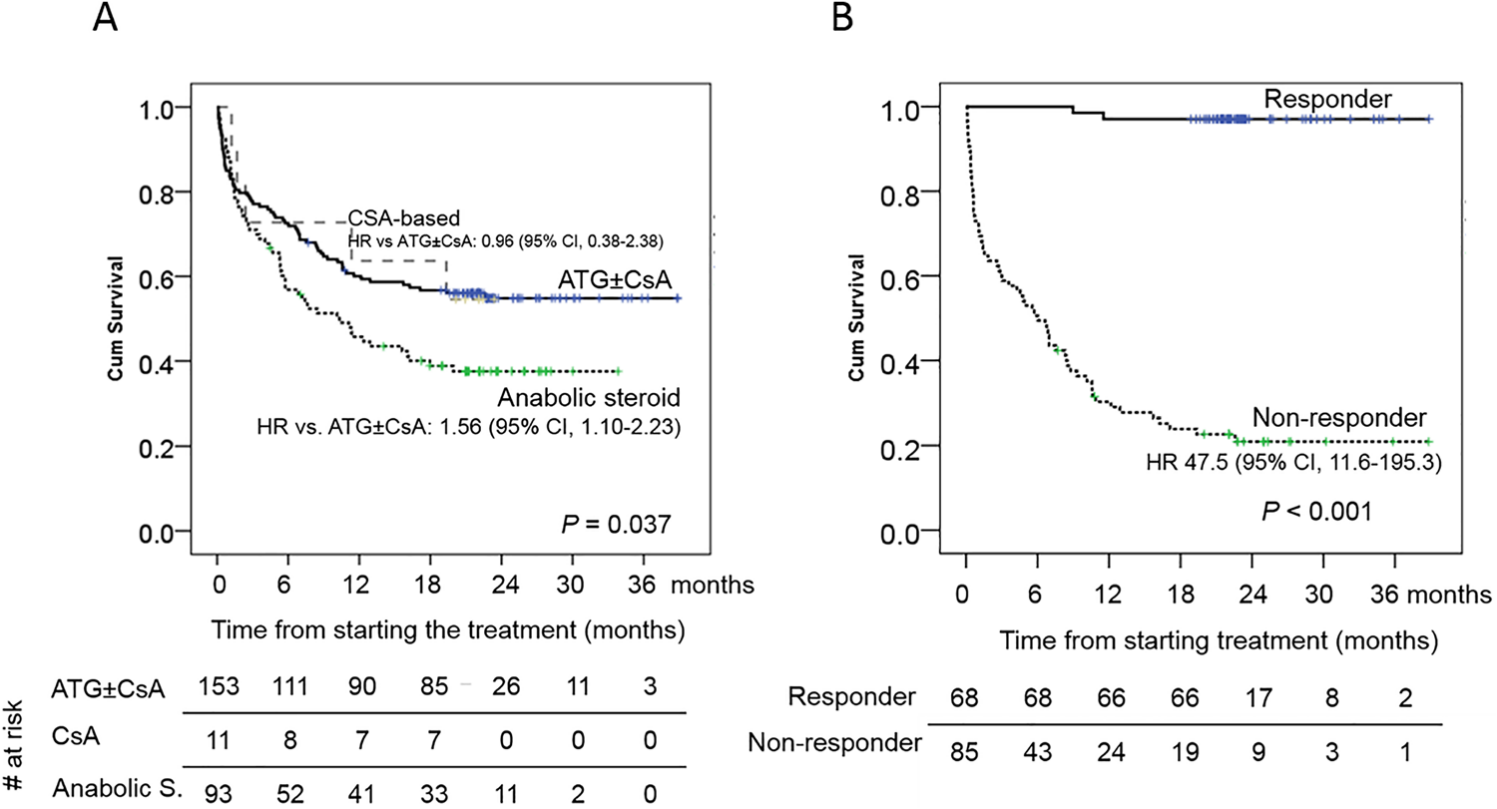
Kaplan–Meier plots of overall survivals among patients with SAA/VSAA (*n* = 280), according to treatment modalities (**A**), and response to rATG ± CsA (*n* = 153, **B**)

The 2-year OS of patients treated with rATG ± CsA, CsA-based treatment, anabolic steroids, and non-specific therapy were 54.8% (95% CI, 50.7–58.9), 54.5% (95% CI, 39.5–69.5), 37.6% (95% CI, 32.5–42.7), and 0% (*P* < 0.001), respectively (Fig. 4A). rATG ± CsA provided a superior OS over anabolic steroids (hazard ratio; HR 1.56, 95% CI, 1.10–2.23, *P* = 0.013) and non-specific treatment (HR 6.83, 95% CI, 4.11–11.36, *P* < 0.001). From multivariate analysis, age > 60 years (HR 1.63, 95% CI, 1.14–2.33, *P* = 0.007), VSAA (HR 2.24, 95% CI, 1.45–3.46, *P* < 0.001), and non-specific treatment (HR 4.96, 95% CI, 2.88–8.54, *P* < 0.001) were independently associated with inferior OS among patients with SAA/VSAA (Table S4B). Notably, patients who attained response after rATG ± CsA had a superior OS than non-responder with HR of 47.5 (95% CI, 11.6–195.3, *P* < 0.001) (Fig. 4B).

## Discussion

In this nationwide prospective study of adult AA patients diagnosed from 2014 to 2016, the annual incidence of adult AA in Thailand was 4.6 per million, which considerably is more or less similar to 3.0–5.0 per million in the previous population-based study in Thailand conducted 20 years ago [6]. Similarity in the annual incidence is also noted among regional parts: 2.8 per million in the southern region (vs 3.0 per million in Songkla and nearby provinces) and 5.6 per million in the north-eastern region (vs 5.0 per million in Khon Kaen and nearby provinces) [6]. The steadiness of the incidence through decades, even the changes in lifestyle and environment toward urbanization, favors the genetic predisposition of autoimmunity as the disease etiology [19, 20]. Nevertheless, the environmental exposer may have an impact on the variation of geographical incidence as the lowest was in the central region (2.5 per million), whereas the incidence was higher in the north-eastern region, the largest agricultural area (5.6 per million) and the peak was in the eastern region, the major industrial district (6.5 per million). The AA annual incidence of 4.6 per million being reported here is approximately twice higher than that from Western countries (ranged 1.5–2.3) [1–5] but is similar to the reports from Asia: 4.8 in Malaysia [21], 5.7 in Taiwan [9], and 7.5 in China [8, 22].

The current study demonstrated a remarkable age-related distribution with steeply increased incidence after the age of 50 (6.2 per million) with a peak incidence at the age over 80 years old (14.3 per million). The pre-existing clonal hematopoiesis, causing bone marrow failure with overlapping features of hypocellular MDS and AA, may lead to a higher incidence of AA among elderly patients [23, 24]. Due to a high proportion of patients with SAA/VSAA in this study (83%) and half of them older than 60 years, the 2-year OS was only 48%, compared with the other reports from Sweden (5-year OS 90.7%) [5], Taiwan (5-year OS 60%) [9], and Spain (2-year OS 57%) [3]. Following the previous population-based studies [3, 5, 9], older age and severity of the disease were the strong predictors of inferior survival among patients with AA.

HSCT is recommended treatment for younger patients of less than 40–50 years old. However, not all eligible patients could undergo this expensive procedure. Furthermore, the government supports only specific number of patients. Therefore, only small number of patients underwent HSCT in this cohort.

rATG is the mainstay of treatment for SAA in Thailand as well as in many countries in Asia. For patients with SAA/VSAA in this cohort, the ORR was considerably low (44.4%), compared with previously published reports of rATG [14, 16]. However, when considering the response among the evaluable patients, the response gradually increased over time with the ORR of 44% at 6 months and 68% at 1 year, consistent with the reports of rATG from Asia (ORR 17% at 6 months and 64% at 12 months) and European Blood and Marrow Transplant (EBMT) (ORR 52% at 6 months and 64% at12 months) [14, 16]. Although the response to rATG in this study was delayed, it was increased over time and the favorable response of rATG was a surrogate marker of long-term survival, similar to previous reports [14, 25].

Anabolic steroids have long been used to treat AA. Its mechanisms include increasing telomerase (TERT) gene expression in hematopoietic cells [26], stimulating erythroid progenitor [27], and enhancing regulatory T-cell [28]. The responses to anabolic steroids are varied, ranging from 25 to 56%, depending on the severity of AA and the treatment combination [29–32]. In this cohort, the response to anabolic steroids appeared to improve over time with an acceptable response in patients with NSAA (40.8%). In SAA/VSAA, the responses were unfavorable (31.2%) compared with rATG ± CsA (44.4%). Giving the inferior OS among patients treated with anabolic steroids, anabolic steroids should therefore be reserved for patients with NSAA and those with SAA/VSAA who are unfit for IST and HSCT.

The outcomes of this study represented the real-world situation of patients with AA. The cases were collected from 30 hospitals, including university and public hospitals, located in all regions of the country; therefore, the data would reasonably reflect the actual AA incidence in Thailand. The further strengths of this study included the prospective design and centrally reviewed diagnosis. However, this study had some limitations. First, the incidence of NSAA may be underestimated especially non-transfusion dependent AA and asymptomatic cases. Second, there was a proportion of non-evaluable patients due to death and loss to follow-up. Third, this study was not randomized, and the comparison between treatment modalities should therefore be interpreted with caution as there would be a high selection bias for older and unfit patients to receive anabolic steroids or supportive care.

In conclusion, the incidence of adult AA in Thailand from this nationwide population-based study is higher than in Western countries, and even higher among the elderly. The real-world outcome of patients with SAA, especially in those aged over 60 years, is substantially poor. The appropriate therapeutic options, as well as the accessibility to advanced treatment, are needed to ensure a better outcome of patients.

## Supplementary Information

Below is the link to the electronic supplementary material.

Supplementary file1 (DOCX 49 KB)

## Data Availability

Yes.
