# Supplementary material for: Adult aplastic anemia in Thailand: incidence and treatment outcome from a prospective nationwide population-based study
Source: Ann Hematol. 2021 Jul 16;100(10):2443–52. doi: 10.1007/s00277-021-04566-0 (PMC8440252; doi:10.1007/s00277-021-04566-0)
Supplement: Supplementary file 1 — Supplementary file1 (DOCX 49 KB) [file 277_2021_4566_MOESM1_ESM.docx]

**Table S1:** Age-Standardized Incidence Rates (ASR) by Geographical Regions

| **Rate per million inhabitants per year** | | | | | | | | |
| --- | --- | --- | --- | --- | --- | --- | --- | --- |
| Region | Population | NSAA  (N = 57) | | SAA/VSAA  (N = 265) | | | Overall  (N = 322) | |
|  |  | Number | ASR | | Number | ASR | Number | ASR |
| Central | 8,261,850 | 3 | 0.4 | | 19 | 2.2 | 22 | 2.6 |
| Northern | 11,664,160 | 16 | 1.3 | | 28 | 2.3 | 44 | 3.6 |
| North Eastern | 32,663,084 | 26 | 0.8 | | 155 | 4.8 | 181 | 5.6 |
| Western | 5,192,892 | 3 | 0.6 | | 17 | 3.2 | 20 | 3.8 |
| Eastern | 6,181,065 | 8 | 1.4 | | 31 | 5.2 | 39 | 6.6 |
| Southern | 5,883,899 | 1 | 0.2 | | 15 | 2.7 | 16 | 2.8 |

NSAA, non-severe aplastic anemia; SAA, severe aplastic anemia; VSAA, very severe aplastic anemia

**Table S2:** Baseline Characteristics of 342 Patients with Aplastic Anemia

|  | **Non-Severe (N=59)** | **Severe (N=238)** | **Very severe (N=45)** | **Total (N=342)** |
| --- | --- | --- | --- | --- |
| **Gender, n (%)** | | | | |
| - Male | 24 (40.7) | 129 (54.2) | 24 (53.3) | 177 (51.8) |
| - Female | 35 (59.3) | 109 (45.8) | 21 (46.7) | 165 (48.3) |
| **Age (years)** | | | | |
| Median (IQR)* | 62 (17-93) | 60 (15-92) | 53 (15-82) | 59 (15-93) |
| **Presenting symptoms, n (%) †** | | | | |
| Anemia | 54 (89.8) | 216 (90.8) | 37 (82.2) | 306 (89.5) |
| Bleeding | 24 (40.7) | 141 (59.2) | 36 (80.0) | 201 (58.8) |
| Infection | 7 (11.9) | 40 (16.8) | 20 (44.4) | 67 (19.6) |

* IQR: interquartile range; **†:** data not mutual exclusive

**Table S3: Treatment Responses to Anabolic Steroids in Patients with NSAA**

| **Total N = 49** | **Month 3** | **Month 6** | **Month 12** | **Month 24** |
| --- | --- | --- | --- | --- |
| Overall response, n (%)  (95% CI) | 14 (28.6%)  (16.6%-43.3%)) | 17 (34.7%)  (21.7%-49.6%) | 20 (40.8%)  (27.0%-55.8%)) | 17 (34.7%)  (21.7%-49.6%) |
| Overall response among evaluable patients, n (%)  (95% CI) | 14/32 (43.8%)  (26.4% - 62.3%) | 17/32 (53.1%)  (34.7% - 70.9%) | 20/29 (71.4%)  (51.3% - 86.8%) | 17/22 (77.3%)  (54.6% - 92.2%) |
| Complete Response, n | 0 | 0 | 1 | 3 |

**Table S4** Univariate and Multivariate Regressions on Clinical Response (**Table S4A**) and Survival Outcome (**Table S4B**) of 280 Patients with SAA/VSAA

**Table S4A**

| **Clinical Response Outcome (n = 280)** | | | | | | |  | | | |
| --- | --- | --- | --- | --- | --- | --- | --- | --- | --- | --- |
|  |  | N | Non-responder (n = 179)  n (%) | Responder (n = 101)  n (%) | *P*-value | |  |  |  | Odds Ratios for treatment response (95% confidence interval) |
| Sex | | | | | | | Model | | | |
|  | Male | 150 | 95 | 55 (36.7) | 0.8 | |  | Severity | | |
|  | Female | 130 | 84 | 46 (35.4) |  |  |  |  | SAA | Ref. |
| Age (years) | | | | | | |  |  | VSAA | 0.45 (0.19-1.11), *P* = 0.08 |
|  | ≤ 60 | 151 | 92 | 59 (39.1) | 0.3 | |  | Treatment modality | | |
|  | >60 | 129 | 87 | 42 (32.6) |  |  |  |  | ATG-based | Ref. |
|  | Median (IQR) |  | 60 (49-69) | 58 (46-66) | 0.3 | |  |  | Anabolic steroids | 0.57 (0.33-0.98), *P* = 0.04* |
| Severity | | | | | | |  |  | Non-specific | 0.00 (0.00-), *P* = 0.99 |
|  | SAA | 235 | 141 | 94 (40.0) | 0.002** | |  |  | CsA-based | 0.66 (0.18-2.35), *P* = 0.52 |
|  | VSAA | 45 | 38 | 7 (15.6) |  |  |  |  |  |  |
| Treatment modality | | | | | | |  |  |  |  |
|  | ATG±CsA† | 153 | 85 | 68 (44.4) | 0.039* | <0.001** |  |  |  |  |
|  | Anabolic steroids | 93 | 64 | 29 (31.2) |  |  |  |  |  |  |
|  | Non-specific | 23 | 23 | 0 (0.0) |  |  |  |  |  |  |
|  | CsA-based | 11 | 7 | 4 (36.4) |  |  |  |  |  |  |

*, **: significant with *P* < 0.05 and *P* < 0.01, respectively

†Including patients received first ATG+CsA as initial (n=143) and subsequent treatment (n=10)

rATG, rabbit antithymocyte globulin; CsA, cyclosporin A; SAA, severe aplastic anemia; VSAA, very severe aplastic anemia; Ref, reference

**Table S4B**

| **Overall Survival Outcome (n = 280)** | | | | |  | | | |
| --- | --- | --- | --- | --- | --- | --- | --- | --- |
| Univariate Cox Regression | | | | | Multivariate Cox Regression | | | |
|  |  | N | Hazard Ratio (95% confidence interval) | *P*-value |  |  | Hazard ratio (95% confidence interval) | |
| Sex | | | | | Model | | | |
|  | Male | 150 | Ref. |  |  | Age (year) | | |
|  | Female | 130 | 1.24 (0.90-1.71) | 0.18 |  |  | ≤ 60 | Ref. |
| Age (year) | | | | |  |  | > 60 | 1.63 (1.14-2.33), *P* = 0.007** |
|  | ≤ 60 | 151 | Ref. |  |  | Severity | | |
|  | > 60 | 129 | 1.40 (1.02-1.93) | 0.037* |  |  | SAA | Ref. |
|  |  |  |  |  |  |  | VSAA | 2.25 (1.46-3.46), *P* < 0.001** |
| Severity | | | | |  | Treatment | | |
|  | SAA | 235 | Ref. |  |  |  | ATG±CsA | Ref. |
|  | VSAA | 45 | 2.63 (1.78-3.81) | < 0.001** |  |  | Anabolic steroid | 1.28 (0.88 - 1.86), *P* = 0.2 |
| Treatment modality | | | | |  |  | Non-specific | 4.96 (2.88-8.55), *P* < 0.001** |
|  | ATG±CsA† | 153 | Ref. |  |  |  | CsA-based | 0.84 (0.34-2.12), *P* = 0.7 |
|  | Anabolic steroids | 93 | 1.57 (1.10-2.23) | 0.013* |  |  |  |  |
|  | Non-specific | 23 | 6.84 (4.11-11.36) | < 0.001** |  |  |  |  |
|  | CsA-based | 11 | 0.96 (0.39-2.38) | 0.93 |  |  |  |  |

*, **: significant with *P* < 0.05 and *P* < 0.01, respectively

†Including patients received first ATG+CsA as initial (n=143) and subsequent treatment (n=10)

rATG, rabbit antithymocyte globulin; CsA, cyclosporin A; SAA, severe aplastic anemia; VSAA, very severe aplastic anemia; Ref, reference

**Table S5:** Univariate and Multivariate Regressions on Treatment Response and Survival among the 153 Patients Treated with ATG + CsA

| **Treatment Response Outcome (n = 153)** | | | | | | | |
| --- | --- | --- | --- | --- | --- | --- | --- |
| Univariate analysis | | | | | Binary-logistic regression | | |
|  |  | Non-responder  (N = 85) | Responder  n (%)  (N = 68) | *P*-value |  |  | Odds Ratios for treatment response  (95% confidence interval) |
| Sex | | | | |  | | |
|  | Male (n = 83) | 47 | 36 (43.4) | 0.7 |  |  |  |
|  | Female (n = 70) | 38 | 32 (45.7) |  |  |  |  |
| Age (year) | | | | | Model | | |
|  | ≤60 (n = 103) | 51 | 52 (50.5) | 0.03* |  | Age > 60 | 0.50 (0.24-1.03), *P* = 0.06 |
|  | >60 (n = 50) | 34 | 16 (32.0) |  |  | ATG dose (mg/kg/day) |  |
|  | Median (IQR) | 57 (49-65) | 55.5 (39.5-60) | 0.02* |  | ≤ 3.0 | Ref. |
| Severity | | | | |  | >3.0 - < 3.5 | 1.38 (0.55-3.47), *P* = 0.4 |
|  | SAA (n = 136) | 72 | 64 (47.1) | 0.06 |  | ≥3.5 – 3.75 | 1.91 (0.74-4.93), *P* = 0.1 |
|  | VSAA (n = 17) | 13 | 4 (23.5) |  |  |  |  |
| Time from diagnosis to ATG-based treatment (day) | | | | |  |  |  |
|  | Median (IQR) | 27 (14-53) | 34 (20- 56) | 0.2 |  |  |  |
| rATG dose (mg/kg/day) | | | | |  |  |  |
|  | Median (IQR) | 3.3 (3.0-3.5) | 3.5 (3.2-3.5) | 0.02* |  |  |  |
| **Survival Outcome (n = 153)** | | | | | | | |
| Univariate Cox Regression | | | | | Multivariate Cox Regression | | |
|  |  | Hazard ratio (95% CI) | | p-value |  |  | Hazard ratio  (95% confidence interval) |
| Sex | | | |  | Model | | |
|  | Male (n = 83) | Ref. | |  |  | Age |  |
|  | Female (n = 70) | 1.18 (0.74-1.90) | | 0.4 |  | ≤ 60 | Ref. |
| Age (year) | | | |  |  | > 60 | 2.01 (1.22-3.28), *P* = 0.005** |
|  | ≤ 60 (n = 103) | Ref. | |  |  | Severity |  |
|  | > 60 (n = 50) | 1.79 (1.11-2.91) | | 0.017* |  | SAA | Ref. |
|  | age as cont. var. | 1.03 (1.01-1.05) | | 0.001** |  | VSAA | 2.73 (1.44-5.17), *P* = 0.002** |
| Severity | | | |  |  |  |  |
|  | SAA (n = 136) | Ref. | |  |  |  |  |
|  | VSAA (n = 17) | 2.34 (1.25-4.37) | | 0.008** |  |  |  |
| Time from Diagnosis to ATG-based treatment (day) | | 0.99 (0.99-1.001) | | 0.07 |  |  |  |

*, **: significant with *P* < 0.05 and *P* < 0.01, respectively

rATG, rabbit antithymocyte globulin; SAA, severe aplastic anemia; VSAA, very severe aplastic anemia

**Table S6** Grade 3-4 Adverse Events Related to rATG+CSA Treatment (CTCE Version 4.0)

|  | | 2.5-3.0 mg/kg/day  (N =30)  n (%) | 3.01-3.49 mg/kg/day  (N= 69)  n (%) | 3.5-3.75 mg/kg/day  (N = 51)  n (%) |
| --- | --- | --- | --- | --- |
| AEs led to early discontinuation | | 5 (16.7) | 7 (10.1) | 7 (13.7) |
| Common AEs (>2%) | | | | |
|  | Serum sickness | 10 (33.3) | 18 (26.1) | 21 (41.2) |
|  | Febrile neutropenia | 2 (6.7) | 10 (14.5) | 6 (11.8) |
|  | Sepsis | 2 (6.7) | 7 (10.1) | 4 (7.8) |
|  | Hypertension | 0 (0.0) | 9 (13.0) | 4 (7.8) |
|  | Infection | 1 (3.3) | 3 (4.3) | 1 (2.0) |
| Uncommon but serious | | | | |
|  | anaphylaxis | 1 (3.3) | 1 (1.4) | 2 (3.9) |
|  | Cardiac arrest | 0 (0.0) | 0 (0.0) | 1 (2.0) |
|  | Hepatotoxicity | 0 (0.0) | 0 (0.0) | 2 (3.9) |
|  | acute renal failure | 0 (0.0) | 0 (0.0) | 2 (3.9) |

Percentages were calculated using denominators derived from safety analysis dataset

Further analyses of adverse events (AE) in different subgroups (i.e. the subgroups by gender, age (≤ 55 years vs. > 55 years), and severity) showed no differences in frequency of the AE categories in each pair of subgroup comparison, except there was a higher hypertension AE incidence among patients aged > 55 years (13.9%; 10 out of 72 patients) as compared with the age of ≤ 55 years (3.8%; 3 out of 78 patients, p = 0.0003) and there was a higher sepsis AE incidence among those with VSAA (36.8%; 7 out of 19 patients) than SAA (3.8%; 3 out of 130 patients, p < 0.001).
